# Stimulation of triple negative breast cancer cell migration and metastases formation is prevented by chloroquine in a pre-irradiated mouse model

**DOI:** 10.1186/s12885-016-2393-z

**Published:** 2016-06-10

**Authors:** Gina Bouchard, Hélène Therriault, Sameh Geha, Yves Bérubé-Lauzière, Rachel Bujold, Caroline Saucier, Benoit Paquette

**Affiliations:** Centre for Research in Radiotherapy, Department of Nuclear Medicine and Radiobiology, Université de Sherbrooke, 3001, 12e Avenue Nord, Sherbrooke, Québec J1H 5 N4 Canada; Department of Anatomy and Cellular Biology, Faculty of Medicine and Health Sciences, Université de Sherbrooke, Sherbrooke, Canada; Service of Radiation Oncology, Université de Sherbrooke, Sherbrooke, Canada; Department of Pathology, Centre Hospitalier Universitaire de Sherbrooke, Sherbrooke, Canada; Department of Electrical and Computer Engineering, Centre d’imagerie moléculaire de Sherbrooke, Sherbrooke, Québec Canada

**Keywords:** Chloroquine, Inflammation, Invasion, Metastasis, Radiation, Triple negative breast cancer

## Abstract

**Background:**

Some triple negative breast cancer (TNBC) patients are at higher risk of recurrence in the first three years after treatment. This rapid relapse has been suggested to be associated with inflammatory mediators induced by radiation in healthy tissues that stimulate cancer cell migration and metastasis formation. In this study, the ability of chloroquine (CQ) to inhibit radiation-stimulated development of metastasis was assessed.

**Methods:**

The capacity of CQ to prevent radiation-enhancement of cancer cell invasion was assessed in vitro with the TNBC cell lines D2A1, 4T1 and MDA-MB-231 and the non-TNBC cell lines MC7-L1, and MCF-7. In Balb/c mice, a single mammary gland was irradiated with four daily doses of 6 Gy. After the last irradiation, irradiated and control mammary glands were implanted with D2A1 cells. Mice were treated with CQ (vehicle, 40 or 60 mg/kg) 3 h before each irradiation and then every 72 h for 3 weeks. Migration of D2A1 cells in the mammary gland, the number of circulating tumor cells and lung metastasis were quantified, and also the expression of some inflammatory mediators.

**Results:**

Irradiated fibroblasts have increased the invasiveness of the TNBC cell lines only, a stimulation that was prevented by CQ. On the other hand, invasiveness of the non-TNBC cell lines, which was not enhanced by irradiated fibroblasts, was also not significantly modified by CQ. In Balb/c mice, treatment with CQ prevented the stimulation of D2A1 TNBC cell migration in the pre-irradiated mammary gland, and reduced the number of circulating tumor cells and lung metastases. This protective effect of CQ was associated with a reduced expression of the inflammatory mediators interleukin-1β, interleukin-6, and cyclooxygenase-2, while the levels of matrix metalloproteinases-2 and −9 were not modified. CQ also promoted a blocking of autophagy.

**Conclusion:**

CQ prevented radiation-enhancement of TNBC cell invasion and reduced the number of lung metastases in a mouse model.

**Electronic supplementary material:**

The online version of this article (doi:10.1186/s12885-016-2393-z) contains supplementary material, which is available to authorized users.

## Background

Breast cancer is a heterogeneous disease, encompassing a number of distinct biological entities that are associated with specific morphological features and clinical behaviors. Triple negative breast cancer (TNBC) accounts for 10–20 % of all breast carcinomas and is characterized by the absence of estrogen receptor (ER), progesterone receptor (PR) and human epidermal growth factor receptor 2 (HER-2) [1]. Recurrence within 3 years of initial treatment is more likely for this aggressive form of breast cancer and results in a mortality risk two times higher than for non-TNBC patients [2]. Without any targeted therapies for TNBC, a better understanding and optimization of adjuvant treatment as radiotherapy remains essential.

Although radiotherapy is recommended to prevent locoregional relapse, the early recurrence found in some TNBC patients suggests that the formation of metastasis is favored in a subgroup of these patients who respond poorly to ionizing radiation. This stimulation of metastasis development could be related to the ability of radiotherapy to trigger an inflammatory response [3]. This inflammation is characterized by an increase of some cytokines and matrix metalloproteinases (MMP) that are known to favor metastasis development [4]. Further supporting this role of inflammatory cytokines, the association between a chronic inflammation and an increased risk of developing several types of cancer, including breast cancer, have been demonstrated [5]. But it is only recently that an acute inflammation induced by radiation in animal models has been associated with breast cancer progression [6, 7]. This feature of radiotherapy may be particularly important since radiation doses used in clinical practice do not always eradicate all cancer cells scattered in the breast. Such doses rather aim at optimizing long-term results with minimal adverse effects. It is therefore important to understand how an inflammation induced by radiation could accelerate the progression of breast cancer.

Enhancement of cancer cell invasion after their irradiation or exposure to free radicals has been reported for pancreatic cancer cells [8], as well as glioma [9], melanoma [10], colon carcinoma [11] and breast cancer cells [12]. These studies were designed to measure the invasiveness of irradiated cancer cells surviving radiation treatment. On the other hand, irradiating healthy tissues surrounding the tumor can also enhance cancer cell invasion. For instance, we showed that pre-irradiation of mouse mammary glands increased the migration of the mouse TNBC cell line D2A1, the number of circulating tumor cells, and favored the development of lung metastases [7]. Similarly, stimulation of cancer cell migration associated with inflammatory mediators has been reported after irradiation of a mouse thigh and a rat brain [6, 13], demonstrating that certain inflammatory mediators stimulate the invasion of cancer cells which enter into the bloodstream and metastasize. These opposite effects of radiation, i.e. kill cancer cells or stimulate their invasiveness, could be particularly important for the TNBC subgroup that is at higher risk of early recurrence [14].

In the present study, we have determined whether administration of chloroquine (CQ) could prevent radiation-stimulated metastasis development in Balb/c mice. CQ is a large spectrum inhibitor used as antimalarial, anti-angiogenesis, autophagy inhibitor and anti-cancer drug [15]. It is also widely used as an anti-inflammatory agent for the treatment of rheumatoid arthritis and lupus erythematous [16, 17]. Because of the importance of inflammation in radiation-enhancement of breast cancer cell invasion, D2A1 mouse mammary carcinoma cell line was chosen instead of human xenografts tumors which require immunodeficient animals. The right third mammary gland of the mouse was irradiated prior the implantation of TNBC cells in order to better isolate the protective effect of CQ against radiation-induced inflammation in healthy tissue. Our study shows that CQ prevented the radiation-stimulated migration of D2A cancer cells in pre-irradiated mammary glands and reduced the development of lung metastases. As regular nonsteroidal anti-inflammatory drugs are usually prohibited during radiation therapy because of potential bleedings [18], CQ could be an interesting option as anti-inflammatory drug, to optimize the effects of this adjuvant treatment.

## Methods

### Cell culture

The TNBC cell lines D2A1, 4T1 and MDA-MB-231 and the non-TNBC cell lines MC7-L1, and MCF-7 were studied. The mouse breast carcinoma D2A1 cells, kindly provided by Dr. Ann F. Chambers (University of Western Ontario, London, ON, Canada), were derived from a spontaneous mammary tumor in a Balb/c mouse [19]. The mouse mammary carcinoma cell line MC7-L1 was generously provided by Dr Alfredo A. Molinolo of the Instituto de Biologia y Medicina Experimental, Concejo Nacional de Investigaciones Cientificas y Técnicas en Facultad de Medicina, Universidad de Buenos Aires, Buenos Aires, Argentina [20]. Other cell lines were purchases from American Type Culture Collection (ATCC, Manassas, VA, USA). We confirmed the TNBC status of the D2A1 cells in collaboration with a pathologist of our institution pathology service using the clinical standard for immunohistochemistry protocols. Antibodies against ER and PR were used as well as Herceptest™ for HER-2, all purchased from Dako (Burlington, ON, Canada). The receptor status for the 4 T1, MDA-MB-231, MC7-L1 and MCF-7 cell lines were already reported (Table 1).

**Table 1:** TNBC status of the breast cancer cell lines

Cell lines	Species	Triple negative	References
MC7-L1	Mouse	No	[20]
4T1	Mouse	Yes	[44]
D2A1	Mouse	^a^ Yes	Additional file 5: Figure S5
MCF-7	Human	No	[45, 46]
MDA-MB-231	Human	Yes	[46]

^a^TNBC status for the cell line D2A1 was determined as described in Materials and Methods

All cell lines were maintained in a 5 % CO_2_ humidified incubator at 37 °C in Dulbecco modified Eagle’s medium (DMEM) (Sigma-Aldrich, Oakville, ON, Canada) supplemented with 10 % fetal bovine serum (Wisent, St. Bruno, QC, Canada), 2 mM glutamine, 1 mM sodium pyruvate, 100 units/ml penicillin and 100 μM streptomycin.

Stable cell population of D2A1 encoding for the fluorescent ubiquitinated-based cell cycle indicator (FUCCI) proteins^33^ were generated as previously described [7].

### In vitro effect of CQ on cell growth and invasion capabilities

Effect of CQ on growth of the MC7-L1, 4T1, D2A1, MCF-7 and MDA-MB-231 cell lines was assessed. Cells (2.5 × 10^4^) plated in 35 mm Petri dishes were either treated with medium (vehicle), 2.5 μM or 5 μM CQ, and their number was determined with a haemocytometer 24, 48 and 72 h later. The experiment was realized in triplicate and repeated 3 times.

For the invasion assay, conditioned media from irradiated Balb/c 3T3 fibroblasts were used as chemoattractant as previously described [7, 12]. Briefly, Balb/c 3T3 fibroblasts seeded in 24-well plates were irradiated using a ^60^Co source (Gammacell 220, Nordion, Canada) at a dose of 5 Gy. Media were immediately removed after irradiation and replaced with DMEM supplemented with 0.1 % BSA and CQ. Twenty-four hrs later, the conditioned media were isolated and used as chemoattractant in the lower compartment of invasion chambers (Becton Dickinson Biosciences, Bedford, MA, USA). Cancer cells were added to the upper compartment in DMEM 0.1 % BSA supplemented with CQ. Cancer cells that crossed the layer of Matrigel™ were fixed 6 h (D2A1, 4T1) or 24 h later (MDA-MB-231, MCF-7, MC7-L1), stained with crystal violet and counted under the microscope. Results were reported as radiation-enhancement ratio. Each experiment was performed in triplicate and repeated two times.

### Mammary gland pre-irradiation and implantation of D2A1 FUCCI cells

The experimental protocols were approved by the Université de Sherbrooke Ethics Committee for Animal Care and Use in accordance with guidelines established by the Canadian Council on Animal Care (Protocol ID number 013–14). An immunocompetent mouse model was preferred to human tumor xenografts implanted in nude mice in order to preserve the inflammatory response induced by radiation. Female retired breeder Balb/c mice (18 to 24 week-old) were obtained from Charles River (Raleigh, NA, USA). Animals were anesthetized with 3 % isoflurane and then immobilized with a stereotactic mice frame adapted to dock on to a Leskell Gamma Knife® Perfexion™ (Elekta, Stockholm, Sweden). The third right mammary gland was irradiated daily with 4 fractions of 6 Gy (dose rate of 1.33 Gy/min) as previously described [7]. To determine whether pre-irradiation of the mammary gland stimulated the migration of mouse mammary cancer cells, D2A1 FUCCI-expressing cells (1 × 10^6^/100 μl PBS) were implanted 3 h after the last irradiation into the pre-irradiated (right side) and non-irradiated (control, left side) mammary glands. Mouse mammary carcinoma cells were also implanted into the mammary glands of sham-irradiated mice to analyze circulating tumor cells and lung metastases that were compared with pre-irradiated animals. Tumor volumes were measured every 3 days according to Balin-Gauthier et al. method [21]. Each experiment was performed in triplicate and repeated at least two times. In another group of animals, mice were euthanized to quantify pro-invasive molecules in mammary glands at different times post-irradiation.

### CQ treatment

CQ purchased from Sigma-Aldrich (C6628, Oakville, Ontario, Canada) was injected intraperitoneally (i.p.) in Balb/c mice at 40 or 60 mg/kg suspended in 0.9 % saline 3 h before each irradiation. Treatment was then administered every 72 h, which corresponds to the half-life of CQ, until euthanasia on day 21. Another group of mice were injected with saline 0.9 % and used as non-treated control.

### Quantification of circulating tumor cells

Blood samples were collected from the lateral saphenous vein of the sham and pre-irradiated mice, treated with vehicle or CQ at day 7 after the injection of D2A1 FUCCI-labeled cells into the mammary glands. Samples diluted 1:10 in PBS were spread in a Petri dish and covered with a glass cover slip. The presence of circulating tumor cells in each blood sample was quantified by fluorescence microscopy from 5 images of representative areas (magnification × 100). Fluorescence microscopy method was chosen instead of FACS analysis because repeated quantifications with small blood samples can be performed in the same animals.

### In vivo and in situ optical imaging

Migration of D2A1 FUCCI-expressing cancer cells in the mammary gland was monitored with an animal optical imager (QOS® Imager, Quidd S.A.S., Val de Reuil, France). Mice were anesthetized with ketamine/xylazine (87 : 13 mg/ml at 1 mg/kg). Bright field images of the mice were taken and then the appropriate filters were selected for red and green fluorescent image acquisition (mKO2, λ_*ex*_ = 472/30, λ_*em*_ = 536/40; mAG, λ_*ex*_ = 531/40, λ_*em*_ = 593/40). The three images were merged for future analysis. Distances of D2A1 cell migration in irradiated and non-irradiated mammary glands were measured to determine the radiation-enhancement ratio, and the protective effect of CQ. Migration was quantified with ImageJ (NIH, USA) as the distance from the nipple (physical landmark for the injection site) to the end of fluorescent smear. Animals were sacrificed on day 21 and tumor and lung specimens were removed. Fluorescence images of the lungs were acquired and the number of metastases was quantified. The diameter of the metastases was also measure using ImageJ. All quantifications were done for sham and irradiated mice, treated with vehicle, 40 mg/kg or 60 mg/kg CQ. Results are from 2 to 3 independent experiments, each realized in triplicate.

### Histology

Mammary tumors and lung specimens containing D2A1 FUCCI-expressing cancer cells were collected and immediately frozen in a solution of Optimum Cutting Temperature (OCT; Electron Microscopy Sciences, Hatfield, PA, USA) or fixed with 4 % paraformaldehyde for pathological examination using H&E staining by the Histology, Electron Microscopy and Phenotyping Services of Université de Sherbrooke. Invasion ratios were quantified on H&E staining using Nanozoomer Digital Pathology software. Cryosections of 3 or 7 μm were made using a Leica CM3050 Microsystems cryostat (Leica Microsystems Inc., Concord, ON, Canada). Slides were dried for 30 min at 37 °C and then stored at −80 °C until further use. The fluorescence emitted by the D2A1 cells was recorded using a FSX100® Bio Imaging Navigator microscope (Olympus, Center Valley, PA, USA) equipped with band pass filters (Chroma Technology Corp, Bellows Falls, VT, USA) for fluorescein isothiocyanate (FITC; λ_*ex*_ = 480/30, λ_*em*_ = 535/40) or tetramethylrhodamine isothiocyanate (TRITC; λ_*ex*_ = 560/40, λ_*em*_ = 630/60). To calculate the ratio of red and green fluorescence intensity of tumors cells, the entire slide was scanned (magnification × 42) and every image was quantified for red and green signals.

### Immunohistochemistry

Immunohistochemistry assays were performed on tumor frozen sections (7 μm) to detect the CD31 blood vessel marker (dilution 1:100; Santa Cruz Biotechnology, Santa Cruz, CA, USA). An anti-goat secondary antibody conjugated with horseradish peroxidase was used for revelation (dilution 1:3000; Cedarlane, Burlington, ON, Canada) combined with the Dako EnVision HRP system (Burlington, ON, Canada). Tissues were counterstained with methyl-green. For each tissue, images of 10 representative areas were taken (magnification × 200) for signal quantification. The number of stained pixels were quantified using Pham et al. method [22] adapted by the Plateforme d’Analyse et de Visualization d’Images (PAVI) of the Université de Sherbrooke. The CD31 area (%) was calculated as the sum of CD31 stained pixels on the total pixels of each image × 100 and reported as radiation-enhancement ratios. Apoptosis in frozen tumor sections (3 μm) was quantified with an ApopTag® peroxidase in situ apoptosis detection kit (EMD Millipore, MA, USA) according to manufacturer’s instruction. The percentage of positive cells was quantified in 10 representative areas (magnification × 200) for each tumor section. The results were reported as percentage of apoptotic cells.

Cell proliferation was measured by Ki67 marker in tumor paraffin-embedded sections. Tissues were deparaffinized with 3 consecutive baths of xylene and dehydrated with ETOH 95 % and 70 %. Tissues were boiled 3 min in citrate buffer pH 6.0 using a pressure cooker. Slides were incubated overnight at 4 °C in a humid chamber with primary antibody (1:100, ab15580, Abcam, Toronto, ON, Canada) and then for 1 h at room temperature with secondary antibody (1:1000, LS-C181152, LifeSpan BioSciences, Seattle, WA, USA). Tissues were counterstained with methyl-green, washed with xylene and sealed with Cytoseal™ 60 mounting medium (18006, Electron Microscopy Sciences, Hatfield, PA, USA). The percentage of positive cells was quantified in 10 representative areas (magnification × 200) for each tumor section using Image-based Tool for Counting Nuclei plugin in imageJ software. The results were reported as percentage of positive cells.

### Quantification of inflammatory and pro-migratory factors

The mRNA levels of cyclooxygenase-2 (COX-2), interleukin-1 beta (IL-1β), interleukin-6 (IL-6) and cytosolic phospholipase A2 (cPLA2) were determined by quantitative real-time polymerase chain reaction (qPCR) in irradiated and contralateral non-irradiated mammary glands (*n* = 3) 6 h after the last session of irradiation as previously described [7].

Tissues were homogenized in 150 mM NaCl, 50 mM Tris pH 7.5, 1 % triton, 0.5 % sodium deoxycholate and 0.1 % sodium dodecyl sulfate. MMP-2 and MMP-9 were quantified by zymography, as previously described [6]. Autophagy markers LC3B1, LC3B2 and p62 were quantified by Western blot. Proteins were resolved in 15 % acrylamide gel and transferred to PVDF membrane, which were probed with LC3B1 + LC3B2 primary antibody (1:10 000, PA5-32254, Thermo Scientific, Rockford, IL, USA), p62 (1:1000, ab56416, Abcam, Toronto, ON, Canada) and secondary antibody (1:10 000, LS-C181152, LifeSpan BioSciences, Seattle, WA, USA). The proteins were revealed by ECL Plus detection kit (PerkinElmer, Waltham, MA, USA). Relative intensity of the bands were normalized to beta-actin internal standard using ImageJ Gel Analyze function.

### Statistical analysis

Experimental data are shown as mean ± standard error mean (SEM). Statistical analyses were performed using one-way analysis of variance (ANOVA) with multiple comparisons test. A *P* value of less than 0.05 was considered to be statistically significant. *P < 0.05, **P < 0.01, ***P < 0.001 and ****P < 0.0001.

## Results

### Radiation-stimulated invasion in TNBC cells was blocked by CQ

**Fig. 1:**
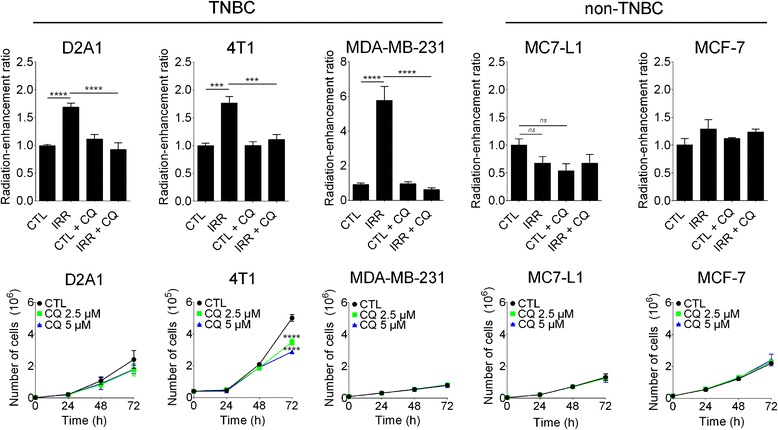
Effect of CQ on breast cancer cell invasion and growth. **a** Conditioned media from irradiated 3T3 fibroblasts was added in the lower compartment of invasion chamber and used as chemoattractant for breast cancer cells added in the upper compartment. Treatment with 5 μM CQ completely blocked radiation-enhancement of invasion in TNBC cell lines. Invasiveness of the non-TBNC cell lines were not modified by the irradiated 3T3 fibroblasts. CTL; Control, IRR; Irradiated, CQ; Chloroquine **b** Effect of CQ at 0, 2.5 or 5 μM on breast cancer cell growth measured 24, 48 and 72 h post treatment. Error bars indicate SEM. The experiment was realized in triplicate and repeated 3 times

The ability of irradiated fibroblasts to increase the invasion of cancer cells was assessed in the TNBC cell lines D2A1, 4T1 (mouse) and MDA-MB-231 (human) and in the non-TNBC cell lines MC7-L1 (mouse) and MCF-7 (human). Used as chemoattractant, conditioned media from irradiated (5 Gy) 3 T3 fibroblasts increased the invasiveness of all TNBC cell lines: D2A1; 1.7-fold (****P < 0.0001), 4T1; 1.8-fold (***P < 0.001) and MDA-MB-231; 5.8-fold (****P < 0.0001), compared to non-irradiated controls. On the other hand, no increase was measured with the non-TNBC cell lines MC7-L1 and MCF-7 (Fig. 1a).

The ability of CQ to prevent this adverse effect of radiation was then assessed; but first, the concentration of CQ that does not modify the growth of these cancer cells was determined. Breast cancer cells were incubated with vehicle, 2.5 or 5 μM CQ and then counted 24, 48 and 72 h later (Fig. 1b). CQ did not significantly decrease the cell proliferation, except for the 4 T1 cell line for which a slower growth was measured for CQ but only after 72 h of incubation (CQ 2.5 μM; ****P < 0.0001, CQ 5 μM; ****P < 0.0001). This late effect was not a constraint since the invasion assays were completed in 6 h for this cell line. A concentration of 5 μM of CQ was therefore chosen.

For all the TNBC cell lines, treatment with CQ completely blocked the stimulation of their invasion induced by radiation (Fig. 1a). It is noteworthy that CQ did not significantly reduce their basal invasion level measured without radiation. On the other hand, invasiveness of the non-TNBC cell lines MCF-7 and MC7-L1, which was not enhanced by irradiated fibroblasts, was also not significantly modified by CQ.

### Inhibition of D2A1 TNBC cell migration in mouse mammary gland

**Fig. 2:**
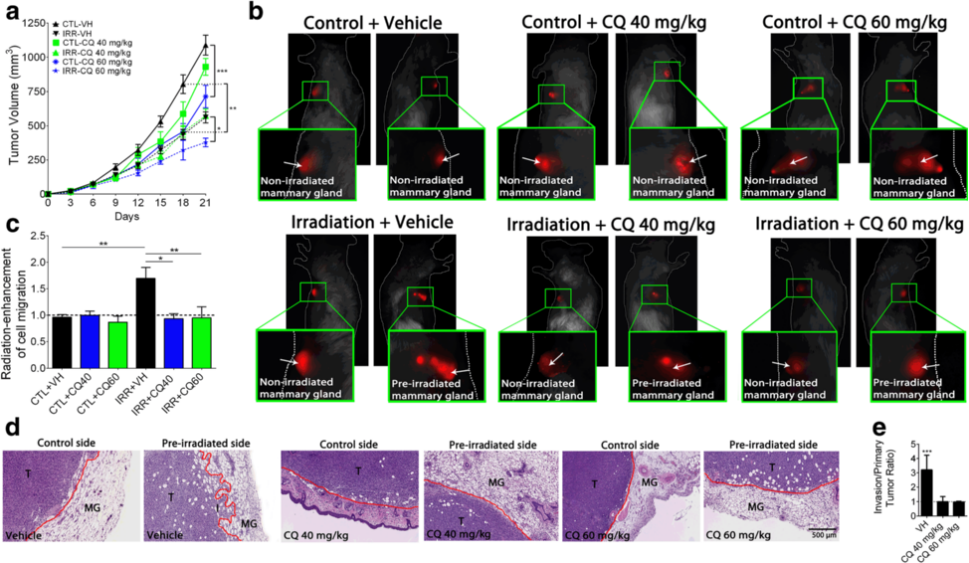
Effect of CQ on D2A1 tumor growth and migration. **a** D2A1 tumor volumes measured after implantation in pre-irradiated or non-irradiated mammary glands of animals treated with vehicle or CQ. Treatment with CQ at 60 mg/kg significantly reduced the tumor volume from day 18 in non-irradiated animals, and from day 21 in tumors implanted in pre-irradiated mammary glands. **b** and **c** in vivo optical imaging of D2A1 cells in mice mammary glands. White arrows = injection site of D2A1 cells. Cell migration in pre-irradiated mammary glands was enhanced by 1.7-fold (**P < 0.01) compared to control side. Treatment with CQ at 40 mg/kg (*P < 0.05) or 60 mg/kg (**P < 0.01) completely blocked radiation-stimulation of cell migration in mammary glands. **d** H&E staining from tumor sections confirming results observed in B and C. T = D2A1 tumor, MG = mammary gland. **e** Quantification of tumor invasion using H&E staining. Invasion was calculated as follow: Invasion area (mm^2^)/Primary tumor area (mm^2^). Results were reported as radiation-enhancement ratio. H&E quantification of tumor sections show a 3.2-fold increase of invasion (***P = 0.004) for tumors implanted in pre-irradiated mammary glands that was completely prevented using CQ

As previously reported, D2A1 tumors implanted in pre-irradiated mammary glands were significantly smaller compared to those in sham-irradiated mammary glands [7]. Treatment with CQ at 40 mg/kg before each session of irradiation, and thereafter at every 72 h, did not further affect tumor growth. The dose of CQ had to be increased to 60 mg/kg to measure a reduction in tumor volume that was significant from day 18 in non-irradiated animals, and from day 21 in tumors implanted in pre-irradiated mammary glands (Fig. 2a). To exclude systemic effect of radiation on tumor growth, tumor volumes of sham-irradiated animals (sham tumors) were compared to control tumors (left side) of pre-irradiated animals as a validation of the mice as its own control in following experiments (Additional file 1: Figure S1).

The effect of CQ on radiation-stimulated migration of D2A1 cells was then assessed. As measured with an animal optical imager, pre-irradiation of the mouse mammary gland increased by 1.7-fold (**P < 0.01) the distance of D2A1 cell migration. This stimulation was completely prevented by treating the animals with CQ at 40 mg/kg (*P < 0.05) or 60 mg/kg (**P < 0.01) (Fig. 2b and c). These results were then confirmed by H&E staining (Fig. 2d and e).

### Reduction of tumor vascularization

**Fig. 3:**
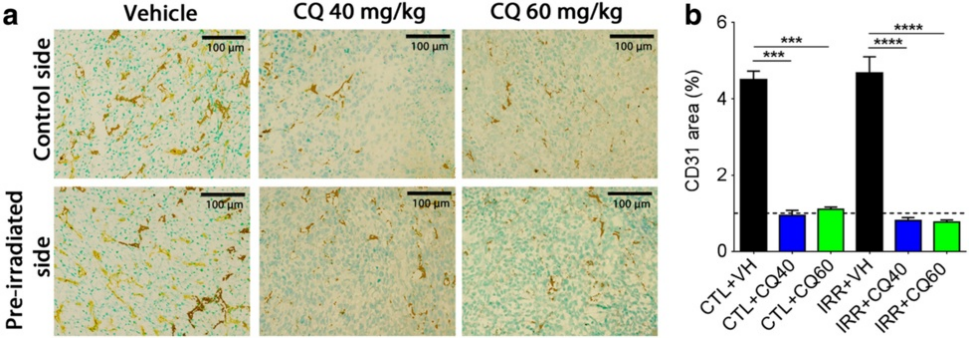
Effect of CQ on tumor vascularization. **a** Immunohistochemistry against CD31 endothelial marker in frozen tumor sections (magnification × 200). **b** Quantification of CD31 signal plotted as percentage of stained area between control (sham) vs control + CQ, or irradiated vs irradiated + CQ. ***P < 0.001, ****P < 0.0001. Error bars indicate SEM for *n* = 3 to 14 independent experiments for each group

Since the anti-angiogenic ability of CQ was previously reported [16], we determined whether this effect of CQ was associated with the inhibition of radiation-enhancement of TNBC cell migration. Pre-irradiation of the mammary gland before implantation of D2A1 tumors did not modify the tumor vascularization compared to tumors implanted in non-irradiated mammary glands, as measured with blood vessel marker CD31. On the other hand, CQ treatment significantly decreased the level of CD31 in tumors implanted in the pre-irradiated and non-irradiated mammary glands (Fig. 3). This reduction was similar for the two doses of CQ studied.

### Effect on cell cycle distribution

**Fig. 4:**
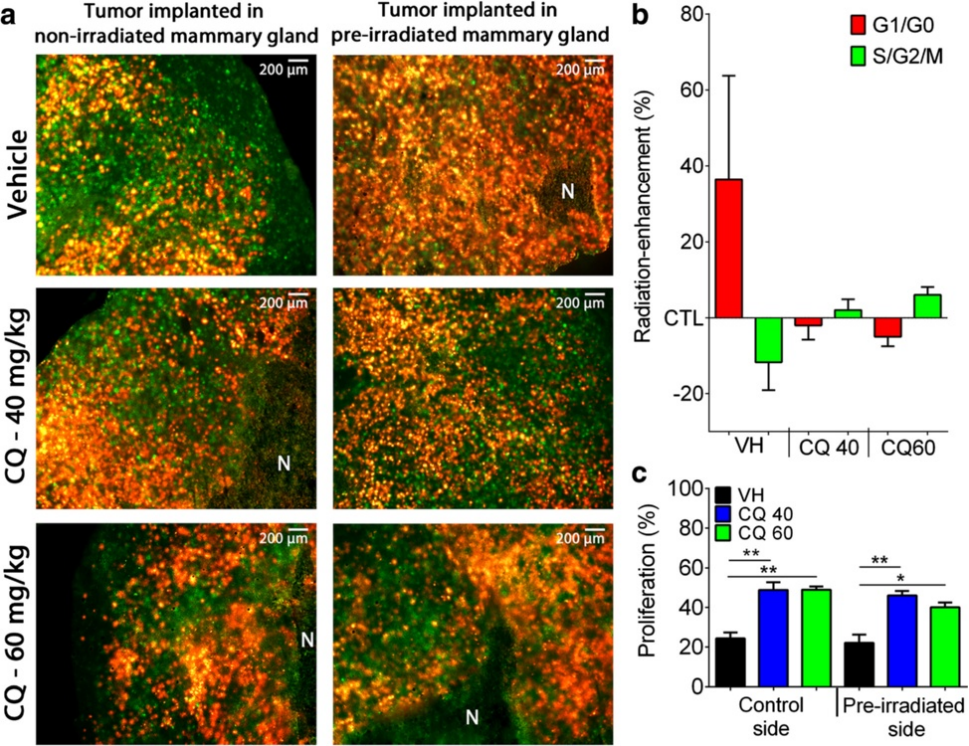
Effect of CQ on cell cycle distribution in D2A1 FUCCI tumors. **a** Representative fluorescence images of frozen sections of mammary tumors used to quantify cancer cells in S/G2/M (green) or G1/G0 (red) phases. **b** Effect of radiation on cell cycle distribution plotted as radiation-enhancement ratio of red and green cells in percentage. **c** Quantification of Ki67 by immunohistochemistry on D2A1 tumor frozen sections. *P < 0.05, **P < 0.01. Error bars indicate SEM for *n* = 4 to 11 independent experiments for each group

In our model, the FUCCI colorimetric vectors expressed by the D2A1 cells generate a green fluorescence when cells are in the S/G_2_/M phases and red fluorescence for the G_1_/G_O_ phases. Using these fluorescent makers, distribution of S/G_2_/M and G_1_/G_O_ phases was determined in frozen sections of tumors implanted in control or pre-irradiated mammary glands. Stimulation of cancer cell migration in pre-irradiated mammary gland was associated with an enrichment of D2A1 cells in G_1_/G_O_ phases (red fluorescence) by 36.4 % and a decrease in S/G_2_/M phases (green fluorescence) by 11.7 %. Treatment with CQ has completely prevented this enrichment in the G_1_/G_O_ phases, as well as the decrease of cells in S/G_2_/M (Fig. 4a and b).

The cell proliferation marker Ki67 was then used to further assess the effect of radiation and CQ on D2A1 cell proliferation. Treatment with CQ at 40 and 60 mg/kg increased by 2-fold the levels of Ki67 expressed in D2A1 tumors (Fig. 4c). Since the Ki67 marker is absent from cells in G0 phase, this suggests that CQ has induced a transfer from quiescent to cycling cell state. Control tumors were also compared with sham tumors to exclude possible radiation-induced systemic bias on proliferation (Additional file 2: Figure S2).

### Reduction of lung metastasis development induced by radiation

**Fig. 5:**
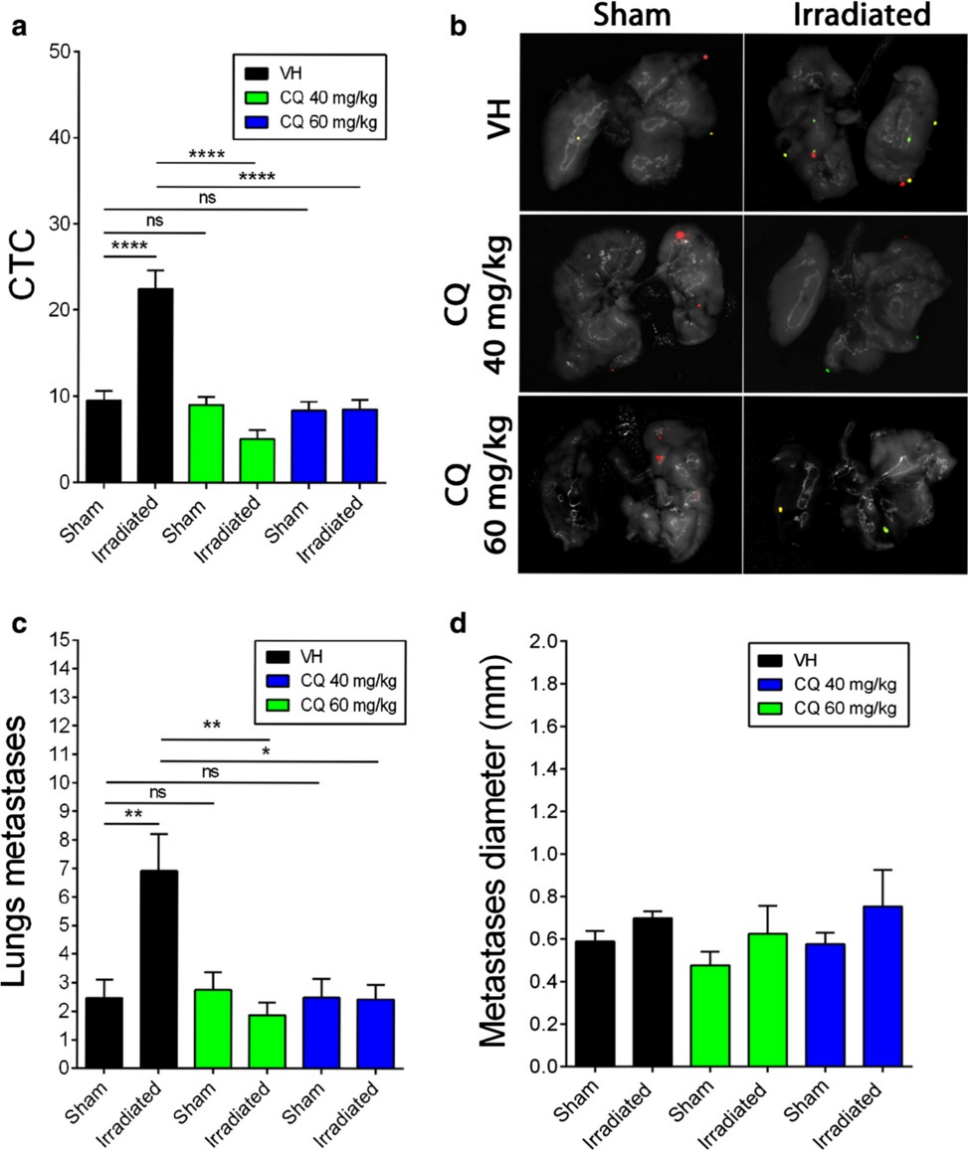
Inhibition of radiation-enhancement of lung metastases with chloroquine. **a** Quantification of circulating tumor cells in blood samples of sham and irradiated mice. **b** Optical imaging of lung metastases. ****P < 0.0001. **c** Quantification of the number of lung metastases. *P < 0.05, **P < 0.01. Sham: Non-irradiated animals with tumor implantation on both sides. Irradiation: Pre-irradiation of the right mammary gland following by tumors implantation on both sides. **d** Quantification of the diameter of lung metastases from optical imaging results. No significant difference was observed for sham or irradiated mice, as for chloroquine treatment. Error bars indicate standard error of the mean (SEM) for *n* = 4 to 15 animals for each group

The preventive effect of CQ on the development of lung metastasis stimulated by radiation was first assessed by quantifying the number of circulating tumor cells (CTC). In the first group of mice, the right mammary gland was pre-irradiated before implantation of D2A1 cells on both sides, while in the second group, the D2A1 cells were also implanted in both mammary glands but in sham-irradiated animals. As we previously reported, pre-irradiation of the mammary gland before the implantation of D2A1 cells increased the number of CTC as well as the number of lungs metastases by 2.4-fold compared to sham-irradiated mice [7]. CQ treatment with 40 mg/kg and 60 mg/kg completely prevented the radiation-enhancement of CTC which came back to the basal level found in sham-irradiated animals (Fig. 5a). Consequently, CQ also prevented the development of lung metastasis induced by radiation (Fig. 5b and c), but did not affect their diameter (Fig. 5d). Interestingly, CQ did not decrease the basal number of lung metastases compared to sham-irradiated animals that received the vehicle. These results suggest that CQ selectively targeted a pathway associated with the radiation-stimulated development of lung metastasis.

### Effect of CQ on apoptosis and autophagy in D2A1 tumors

**Fig. 6:**
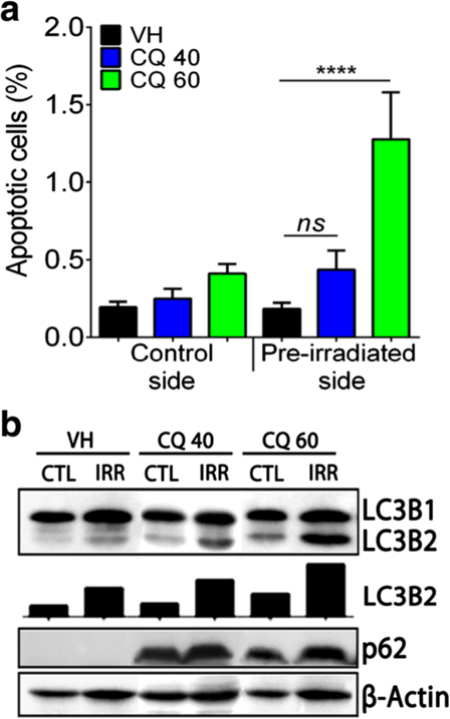
Apoptosis and autophagy analyses of D2A1 tumors. **a** TUNEL assay quantification of the percentage of apoptotic cells in tumor sections of each groups of mice. ****P < 0.0001. Error bars indicate SEM for *n* = 3 to 6 independent experiments. **b** Immunoblot of protein lysates from D2A1 tumors for autophagy markers. Experiment was realized in triplicate

To further assess how CQ prevented the formation of new metastases, apoptosis and autophagy were measured in D2A1 tumors. Treatment with 40 mg/kg of CQ did not significantly modify the percentage of apoptotic cells. An increase by 3-fold compared to vehicle was observed at 60 mg/kg CQ, but only in tumors implanted in pre-irradiated mammary glands (****P < 0.0001) (Fig. 6a).

Quantification of autophagy markers LC3B1 and 2 by Western blot was then performed in tumor homogenates. As expected, the expression of LC3B2 was increased by radiation, supporting an accumulation of autophagosomes. This accumulation was then confirmed to be an increase of autophagy since there is no accumulation of the p62 marker. On the other hand, the blockage of autophagy, preferentially in tumors implanted in pre-irradiated mammary glands, was supported by the accumulation of p62 in CQ-treated tumors, which is usually degraded when autophagy is activated (Fig. 6b and Additional file 3: Figure S3). Radiation-induced systemic bias on autophagy were excluded by comparing autophagy marker in sham and control tumors (Additional file 3: Figure S3 and Additional file 4: Figure S4). Overall, autophagy was preferentially induced in tumors implanted in pre-irradiated mammary glands underlying the importance of tumor microenvironment affecting the tumor.

### Assessment of pro-migratory and inflammatory factors

To characterize these adverse effects of radiation, some pro-migratory and inflammatory factors were quantified in pre-irradiated and control mammary glands. A CQ dose of 40 mg/kg was chosen to exclude the induction of cell death occurring at higher doses.

**Fig. 7:**
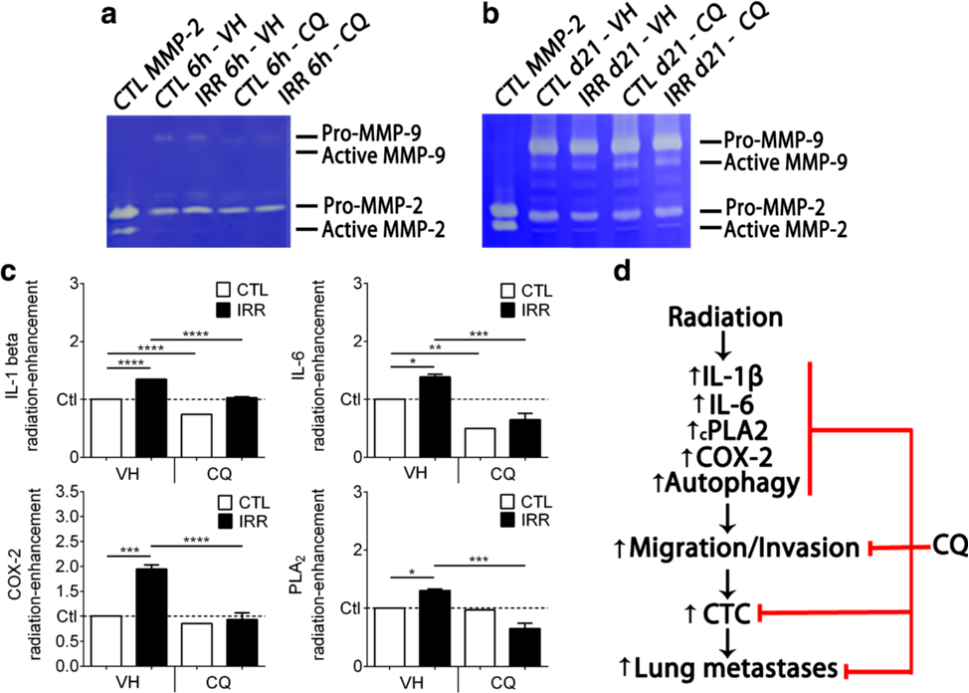
Quantification of pro-migratory and pro-inflammatory factors after chloroquine treatment. **a** Zymogram of MMP-2 and −9 levels after chloroquine treatment performed on protein lysates of both irradiated and non-irradiated mammary glands collected 6 h after irradiation. **b** Zymogram of MMP-2 and −9 levels after chloroquine treatment performed on protein lysates of D2A1 tumors implanted in pre- irradiated and non-irradiated mammary glands collected on sacrifice day (day 21). **c** Effect of chloroquine 40 mg/kg on the relative expression of pro-inflammatory genes potentially in mammary quantified by qPCR 6 h after the last session of irradiation. Relative mRNA expressions are plotted as a radiation enhancement ratio. *P < 0.05, **P < 0.01, ***P < 0.001, ****P < 0.0001. **d S**ummary of the proposed mechanism of chloroquine in the prevention of TNBC invasion stimulated by radiotherapy. Error bars indicate SEM. Experiments were realized in triplicate

The proteases MMP-2 and MMP-9 are known to favor the migration and invasion of cancer cells. Their levels were determined by zymography in mammary glands 6 h after the last irradiation and 21 days after D2A1 tumor implantation (Fig. 7a and b). Radiation did not increase the levels of MMP-2 and −9 in the mammary glands that were implanted/not implanted with the D2A1 tumor. The level of either of these proteases was not reduced after treatment with CQ at 40 mg/kg.

Expression of some inflammatory mediators potentially involved in cancer cell invasion were then quantified (Fig. 7c). The relative mRNA levels of IL-1β and IL-6 were significantly increased 6 h post-irradiation, as measured by qPCR. Regarding the pathway of prostaglandins, a higher expression of COX-2 and cPLA2 were also measured in irradiated mammary glands. Treatment with CQ significantly decreased the expression of IL-1β and IL-6 in both irradiated and non-irradiated mammary glands, and completely inhibited the stimulation COX-2 and cPLA2 induced by radiation.

## Discussion

For the subgroup of TNBC patients that responds poorly to radiotherapy, the risk of recurrence is very high during the first three years after treatment and cure is unlikely [23]. The concept of radiation-stimulated cancer cell migration and invasion is well accepted [24], but the hypothesis suggesting that formation of metastasis could be stimulated by radiation in some TNBC patients still need to be validated. Meanwhile, it has been shown in our previous pre-clinical study that pre-irradiation of a Balb/c mouse mammary gland increased the migration of murine TNBC cells, the number of CTC and favored the development of lung metastases [7]. By irradiating the mammary gland prior to implantation of TNBC cells, this previous study properly demonstrated the contribution of inflammatory mediators released from healthy tissues on metastasis development.

In the present study, we first showed that these adverse effects of radiation were observed in vitro only in the TNBC cell lines and that they can be prevented by CQ. It should be noted that fibroblasts were used to mimic the stroma in invasion chambers but the role of other stromal components in radiation-enhancement of breast cancer cells should not be excluded and requires further investigation. Also, it remains to be determined why radiation did not stimulate the invasion of non-TNBC cancer cells. Also, it is noteworthy that the protective effect of CQ in vitro was not related to inhibition of cancer cell proliferation since no significant effect on cell growth was measured.

Accumulation of CQ in the trans-Golgi network leads to its alkalinization which deregulates the maturation of many proteins, including MMP. MMP-2 and–9 play an important role in cancer cell migration and invasion by cleaving proteins of the extracellular matrix [25, 26]. In the present study, no increase of MMP-2 and −9 was found in irradiated Balb/c mouse mammary gland, and treatment with CQ did not reduce their basal levels. However, a possible involvement of these MMP in breast cancer cell invasion cannot be ruled out since an increased activity of these MMP and a stimulation of cancer cell invasion was observed in other pre-clinical models such as irradiated mouse thigh and rat brain [6, 13]. In breast cancer patients, radiotherapy can increase the plasma level of MMP-9 [27] and the level of MMP-2 was also significantly higher in skin biopsies of women after radiotherapy, relative to non-irradiated skin [28]. On the other hand, reduction of MMP-2 and–9 expression in vitro in the MDA-MB-231 cells was reported at higher doses of CQ than used in our study [29]. Therefore, it remains to be determined in TNBC patients whether radiation can increase the expression of MMP-2 and–9, and whether this can be prevented by CQ.

It was reported that the development of radiation-stimulated lung metastasis after the irradiation of the mammary gland was correlated with inflammatory pathways involving COX-2 as well as IL-1β and IL-6 cytokines [7]. As CQ is also used as an anti-inflammatory agent for the treatment of rheumatoid arthritis and lupus erythematous [16, 17], we determined whether its anti-cancer effect could be associated with a down-regulation of these inflammatory pathways.

In irradiated mouse mammary glands, the stimulation of cPLA_2_ (the first enzyme in the production of prostaglandins) and COX-2 expression were completely prevented by CQ treatment. This inhibitory effect of CQ may have a major impact on breast cancer patient survival. Indeed, elevated expression of COX-2 was associated with poor prognosis and distant metastases in TNBC patients [30, 31], while radiation-enhancement of cancer cell invasion as assessed in vitro can be completely prevented by adding a COX-2 inhibitor [12]. These results support the hypothesis that the inhibition of COX-2 may increase the disease free-survival of TNBC patients, as previously observed for early stage non-TNBC patients [32].

It is noteworthy that CQ did not reduced the basal levels of cPLA2 and COX-2 measured in non-irradiated mammy glands. Since COX-2 is inducible only under pathological or inflammatory conditions, this may suggest that the effect of CQ would be specific to irradiated tissues, resulting in fewer adverse effects for non-irradiated healthy tissues.

We previously reported that the inflammatory cytokine IL-1β was increased in the conditioned media of fibroblasts following radiation. In the same study, IL-1β stimulated the invasiveness of MDA-MB-231 TNBC cells, and this invasive effect was prevented by adding an anti-IL-1β antibody [33]. The resulting enhancement of the invasion appears to be related to an increased expression of COX-2, since the addition of a COX-2 inhibitor completely prevented the stimulation of cancer cell invasion induced by IL-1β [12, 33]. In our mouse model of TNBC, the protective effect of CQ on metastasis development was also associated with a reduction of IL-1β expression, suggesting that this cytokine is a primary target of CQ in the development of lungs metastases.

Regarding IL-6, it is the most important cytokine associated with poor prognosis for breast cancer, and it is known for controlling breast cancer cell growth and regulating cancer stem cell renewal [34]. IL-6 has also been reported to stimulate the proliferation and migration of breast cancer cells in vitro as well as tumor progression [35], but its potential connection with radiotherapy was less studied [34]. Nevertheless, Yu et al. reported that radiation-induced IL-6 in MDA-MB-231cells promoted the invasion and migration of non-irradiated neighboring cells [36]. In our mouse model, CQ reduced the expression of IL-6 in irradiated and non-irradiated mammary glands in the same manner observed with IL-1β suggesting that this cytokine could also be associated with induction of lung metastasis.

Irradiation of healthy tissues surrounding a tumor can modify the balance between proliferation and migration of cancer cells [7, 13]. This migration/proliferation dichotomy was described as mutually exclusive or as a «Go or Grow» phenomenon [37]. Using the FUCCI cell cycle reporter system [38], irradiation of a rat brain or a mouse mammary gland favored the migration of cancer cells and their accumulation in the G_1_/G_0_ phases [7, 13]. This suggests that cytokines released from irradiated tissues could stimulate the migration/invasion of cancer cells through a reduction of their proliferation. Treatment with CQ has successfully reduced the radiation-enhanced accumulation of D2A1 cells in the G_1_/G_0_ phases (red fluorescence), supporting the inhibition of radiation-induced migration in mammary glands. These results are consistent with the decrease of G_1_/G_0_ cells after CQ treatment previously observed in human TNBC cell lines by Jiang et al.[39]. The authors reported the induction of cell cycle arrest in G2/M which may affect the interpretation of cell proliferation with the marker Ki67. This marker of cell proliferation is present in both G2 and M phases. Consequently, an arrest in G2/M may increase the number of Ki67 positive cells, giving the false indication that more tumor cells are proliferating. Indeed, the increased number of Ki67 positive cells measured in our study is expected to be associated with a cell blockage in G2/M rather than an increase of cell proliferation.

The reduction of CTC and the number of lung metastases was not caused by a reduction of tumor blood supply since the presence of CD31 blood vessel marker was not affected by radiation. It was then impossible to associate the protective effect of CQ with the reduction of tumor vascularization.

Our results showed that stimulation of metastasis development stimulated by radiation was inhibited by CQ without affecting the tumor volume. Our results also showed that a low level of apoptosis was only promoted in D2A1 tumors with high dose of CQ (60 mg/kg) in presence of radiation but not with 40 mg/kg of CQ. This suggests that the adverse effect of radiation on the development of metastasis can be prevented by low doses of CQ that would not induce apoptosis in healthy tissues. Consequently, a low systemic toxicity after treatment with CQ could be expected.

CQ is also described as an inhibitor of autophagy. Autophagy is a survival pathway activated in response to stress whereby cellular components are degraded to recycle energy, promote cell survival and cancer resistance. However, if the cells cannot recover from the damage, autophagy will ultimately lead to cell death. Therefore, autophagy could also exert a significant control over the progression of cancer and tissue homeostasis [40]. Our results showed that treatment with CQ blocked autophagy. These findings are consistent with those of Jensen et al. [41], who reported that CQ was highly effective in preventing autophagy. These authors also reported that CQ preferentially accumulated in acidic tumor environment than in normal tissue, suggesting that CQ could be less non-toxic for normal tissues. The increase of autophagy observed in tumor implanted in pre-irradiated tissue could be directly associated to this previous observation. Overall, according to the experimental conditions, autophagy can be either cytotoxic (prolonged autophagy will eventually lead to cell death) or cytoprotective (survival mechanism for the cell). Autophagy is clearly a complex process and its role in TNBC patients remains to be further explored. Without knowing how exactly autophagy was regulated, the preferential blocking of autophagy associated with the accumulation of LC3B2 observed for tumors implanted in pre-irradiated mammary glands seems to be associated with the prevention of the radiation-stimulated of breast cancer cell migration.

Combined with radiation, CQ successfully induced cell death in several human TNBC cell lines [42, 43]. Zhao et al. have shown the radiosensitivity potential of CQ in MDA-MB-231 TNBC cells, by reporting enhanced apoptosis and necrosis [42]. In our study, the mammary gland was irradiated before its implantation with D2A1 cells. Therefore, the anti-cancer effect of CQ cannot be related to a direct radiosensitization but rather to an indirect effect on cancer cells that is mediated by irradiated stroma. The experimental protocol used in this study has provided to confirm that CQ prevents the stimulation of the metastasis development induced by the irradiated stroma. Taken together, these results suggest that treating TNBC patients with CQ could further increase the anti-tumor effect of radiotherapy and reduce the potential adverse effects of radiation-induced inflammation on the stimulation of metastasis development.

## Conclusion

In conclusion, the ability of radiation to stimulate the invasion of cancer cells was observed in vitro only in TNBC cell lines. In our mouse model of TNBC, radiation stimulates the cancer cell migration and development of metastasis which seems to involve multiple inflammatory pathways including those of COX-2, IL-1β and IL-6. These adverse effects of radiation were prevented by treating the animals with CQ. A proposed mechanism is presented in Fig. 7d. Based on these results, a clinical trial to determine whether treatment with CQ could increase the disease-free survival of the TNBC patients that poorly respond to radiation treatment could be undertaken.

## Abbreviations

BSA, bovine serum albumin; CD31, cluster of differentiation 31; Co, cobalt; COX-2, cyclooxygenase-2; cPLA2, cytosolic Phospholipase A2; CQ, chloroquine; CTC, circulating tumor cell; CTL, control; DMEM, Dulbecco modified Eagle’s medium; ER, estrogen receptor; FITC, fluorescein isothiocyanate; FUCCI, fluorescent ubiquitinated-based cell cycle indicator; H&E, haematoxylin and eosin; HER2, human epidermal growth factor receptor 2; HRP, horse radish peroxidase; i.p., intraperitoneal; IL-1β, interleukin-1 beta; IL-6, interleukin-6; IRR, irradiated; LC3, light chain 3; mAG, *monomeric Azami Green*; mKO2, *monomeric Kusabira Orange 2*; MMP, matrix metalloproteinase; OCT, optimum cutting temperature; PR, progesterone receptor; qPCR, quantitative polymerase chain reaction; TNBC, triple negative breast cancer; TRITC, tetramethylrhodamine isothiocyanate; VH, vehicle.

## Additional files

Additional file 1: Figure S1. Validation of the mice as its own control in mice pre-irradiated at the right mammary gland. D2A1 tumor volumes of sham irradiated animals (sham tumors) were compared to control tumors (left side) of pre-irradiated animals. Error bars indicate s.e.m. for *n* = 6 to 15 animals for each group. (TIF 224 kb) Additional file 2: Figure S2. Ki67 immunohistochemistry in sham (nonirradiated animals) and control tumors (left side of pre-irradiated animals) were realized to exclude possible systemic effect of radiation on tumor proliferation. The experiment was realized in triplicate. Sham-VH vs Sham-CQ 40; *P* < 0.0001, Sham-VH vs CTL-CQ 40; *P* = 0.0002, Sham-VH vs Sham-CQ 60; *P* = 0,0001, Sham-VH vs CTL-CQ 60; *P* < 0,0001, CTL-VH vs Sham-CQ 40; *P* < 0,0001, CTL-VH vs CTL-CQ 40; *P* = 0,0002, CTL-VH vs Sham-CQ 60; *P* = 0,0001, CTL-VH vs CTL-CQ 60; *P* < 0,000. Additional file 3: Figure S3. Immunoblot of autophagy markers were realized in sham (non-irradiated animals) and control tumors (left side of pre-irradiated animals) to exclude possible systemic effect of radiation on tumor autophagy. The experiment was realized in triplicate. (TIF 281 kb) Additional file 4: Figure S4. Quantitative densitometry from Western blots of the expression of (A) LCB3I, (B) LCB3II (Sham-CQ 60 vs IRR-CQ 60; *P* = 0.0024, CTL-CQ 60 vs IRR-CQ 60; *P* = 0.0182, IRR-VH vs IRR-CQ 60; *P* = 0.0009) and (C) p62 autophagy markers calculated using ImageJ Gel Analyze function. Additional file 5: Figure S5. Hormonal status of D2A1 cell line was confirmed by immunohistochemistry as described in Materials and Methods. No nuclear (ER and PR) as well as membrane (HER2) staining were observed. D2A1 cells were then revealed to be triple negative by a pathologist of our institution. (TIF 9131 kb)
