# Supplementary figures and images for: Stimulation of triple negative breast cancer cell migration and metastases formation is prevented by chloroquine in a pre-irradiated mouse model
Source: BMC Cancer. 2016 Jun 10;16:361. doi: 10.1186/s12885-016-2393-z (PMC4901430; doi:10.1186/s12885-016-2393-z)

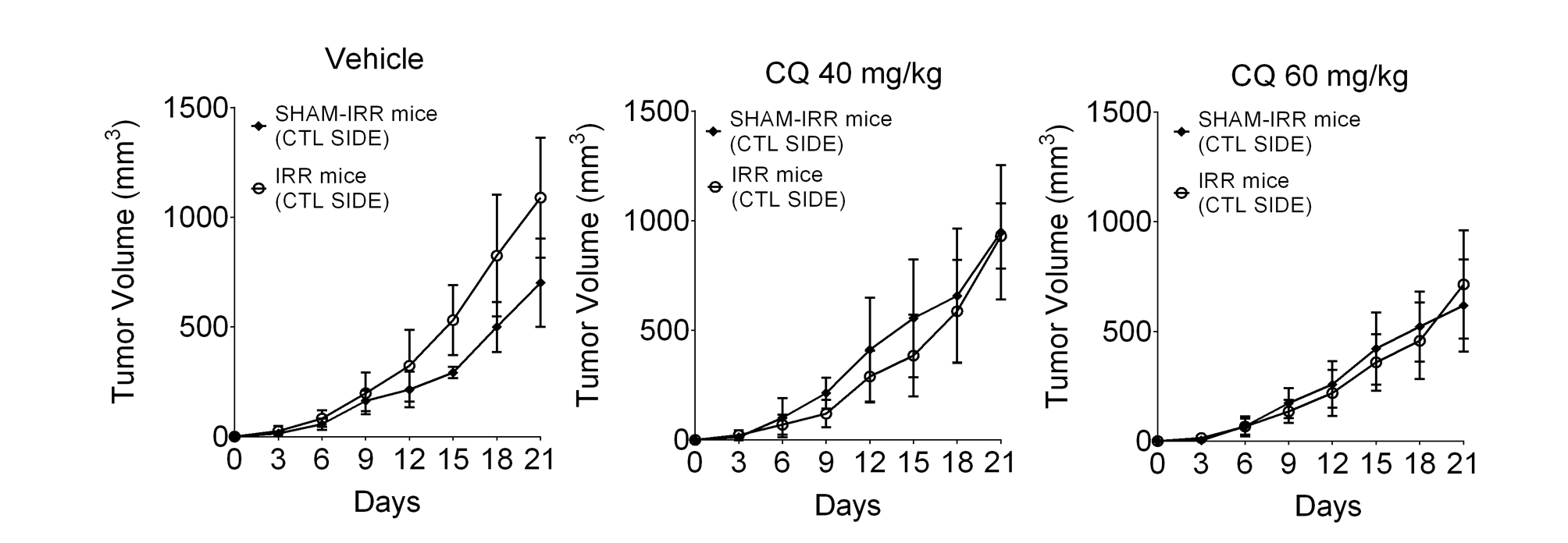

Supplement: Additional file 1: Figure S1. — Validation of the mice as its own control in mice pre-irradiated at the right mammary gland. D2A1 tumor volumes of sham irradiated animals (sham tumors) were compared to control tumors (left side) of pre-irradiated animals. Error bars indicate s.e.m. for n = 6 to 15 animals for each group. (TIF 224 kb) [file 12885_2016_2393_MOESM1_ESM.tif]

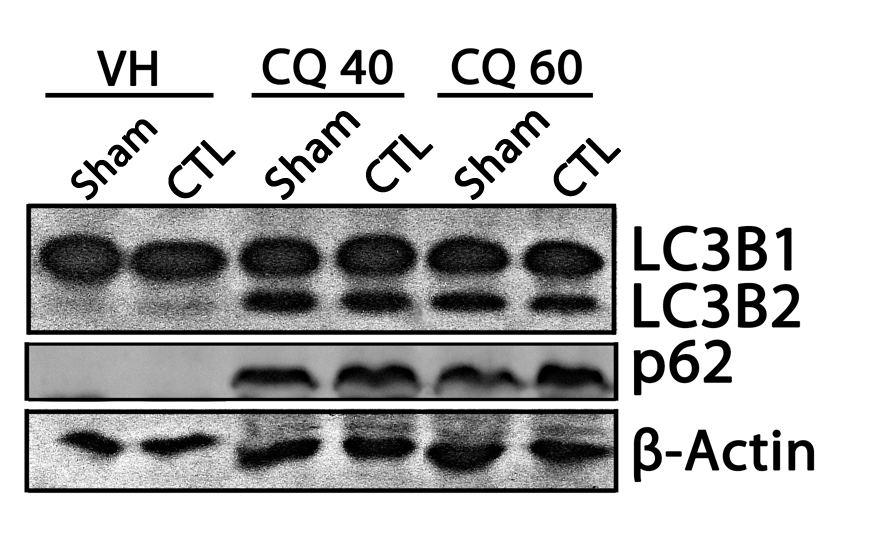

Supplement: Additional file 3: Figure S3. — Immunoblot of autophagy markers were realized in sham (non-irradiated animals) and control tumors (left side of pre-irradiated animals) to exclude possible systemic effect of radiation on tumor autophagy. The experiment was realized in triplicate. (TIF 281 kb) [file 12885_2016_2393_MOESM3_ESM.tif]

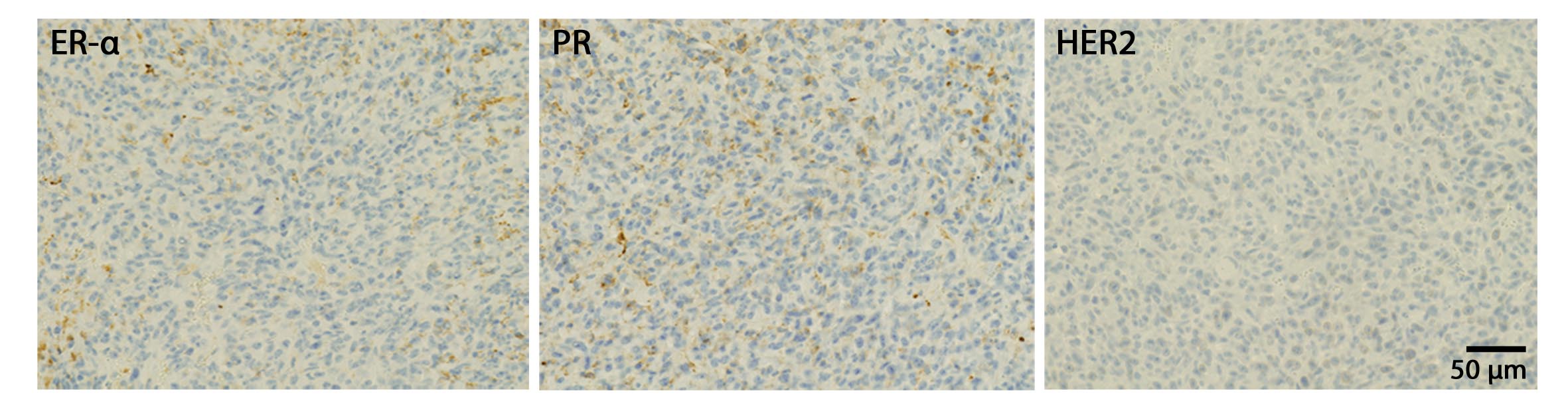

Supplement: Additional file 4: Figure S4. — Quantitative densitometry from Western blots of the expression of (A) LCB3I, (B) LCB3II (Sham-CQ 60 vs IRR-CQ 60; P = 0.0024, CTL-CQ 60 vs IRR-CQ 60; P = 0.0182, IRR-VH vs IRR-CQ 60; P = 0.0009) and (C) p62 autophagy markers calculated using ImageJ Gel Analyze function. [file 12885_2016_2393_MOESM4_ESM.jpg]

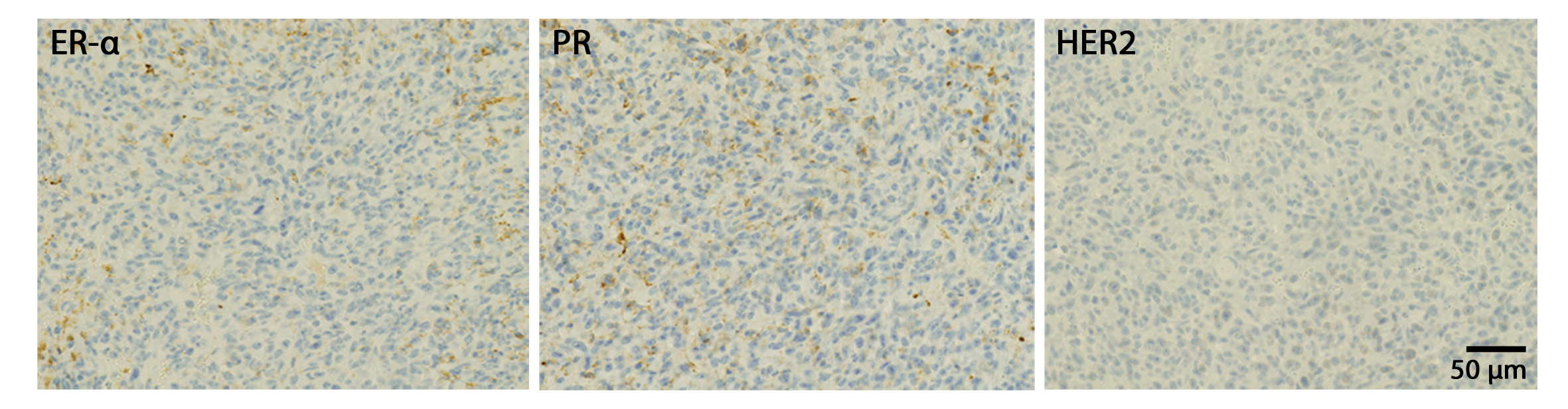

Supplement: Additional file 5: Figure S5. — Hormonal status of D2A1 cell line was confirmed by immunohistochemistry as described in Materials and Methods. No nuclear (ER and PR) as well as membrane (HER2) staining were observed. D2A1 cells were then revealed to be triple negative by a pathologist of our institution. (TIF 9131 kb) [file 12885_2016_2393_MOESM5_ESM.tif]
